# A Wearable Wrist Band-Type System for Multimodal Biometrics Integrated with Multispectral Skin Photomatrix and Electrocardiogram Sensors

**DOI:** 10.3390/s18082738

**Published:** 2018-08-20

**Authors:** Hanvit Kim, Haena Kim, Se Young Chun, Jae-Hwan Kang, Ian Oakley, Youryang Lee, Jun Oh Ryu, Min Joon Kim, In Kyu Park, Hyuck Ki Hong, Young Chang Jo, Sung-Phil Kim

**Affiliations:** 1 Department of Electrical Engineering, Ulsan National Institute of Science and Technology (UNIST), Ulsan 44919, Korea; coreavit7@unist.ac.kr (H.K.); sychun@unist.ac.kr (S.Y.C.); 2 Human Care System Research Center, Korea Electronics Technology Institute (KETI), Seongnam 13509, Korea; chem_is_try87@nate.com (H.K.); hkhong@keti.re.kr (H.K.H.); 3 Department of Human Factors Engineering, Ulsan National Institute of Science and Technology (UNIST), Ulsan 44919, Korea; doskian@unist.ac.kr (J.-H.K.); ian.r.oakley@gmail.com (I.O.); yrlee@unist.ac.kr (Y.L.); 4 Research Institute of H3 System, Daejeon 34036, Korea; apache70@gmail.com (J.O.R.); mjkim@h3system.co.kr (M.J.K.); ikpark@h3system.co.kr (I.K.P.)

**Keywords:** multimodal biometrics, multispectral skin photomatrix, ECG, majority voting, integrated wearable device

## Abstract

Multimodal biometrics are promising for providing a strong security level for personal authentication, yet the implementation of a multimodal biometric system for practical usage need to meet such criteria that multimodal biometric signals should be easy to acquire but not easily compromised. We developed a wearable wrist band integrated with multispectral skin photomatrix (MSP) and electrocardiogram (ECG) sensors to improve the issues of collectability, performance and circumvention of multimodal biometric authentication. The band was designed to ensure collectability by sensing both MSP and ECG easily and to achieve high authentication performance with low computation, efficient memory usage, and relatively fast response. Acquisition of MSP and ECG using contact-based sensors could also prevent remote access to personal data. Personal authentication with multimodal biometrics using the integrated wearable wrist band was evaluated in 150 subjects and resulted in 0.2% equal error rate (EER) and 100% detection probability at 1% FAR (false acceptance rate) (PD.1), which is comparable to other state-of-the-art multimodal biometrics. An additional investigation with a separate MSP sensor, which enhanced contact with the skin, along with ECG reached 0.1% EER and 100% PD.1, showing a great potential of our in-house wearable band for practical applications. The results of this study demonstrate that our newly developed wearable wrist band may provide a reliable and easy-to-use multimodal biometric solution for personal authentication.

## 1. Introduction

Biometrics are a promising tool that can replace or supplement existing knowledge-based or possession-based authentication/identification systems. Biometrics have recently served as one of the key user authentication and/or identification methods as they provide strong security as well as convenience [1,2,3,4,5]. Various biometric technologies have so far leveraged human biological characteristics including fingerprint [6], face [7], iris [8], hand vein [9,10], electroencephalogram (EEG) [11,12], electrocardiogram (ECG) [13,14,15], as well as a recently explored multispectral skin photomatrix (MSP) [16].

To realize practical biometric systems, it is important to satisfy key criteria such as collectability, performance, and circumvention [1]. A number of studies have proposed solutions to meet the criteria: wearable biometric systems can enhance collectability [17], multimodal biometrics with advanced machine learning algorithm can deliver high performance [5,18,19,20], and spoof-resistant schemes for biometrics can overcome circumvention [21]. However, few studies have addressed all of them at once. For instance, Aronowitz et al. proposed multimodal biometrics for mobile devices using voice, face, and chirography with 0.1% equal error rate (EER) in 100 subjects [22]. However, these biometrics are relatively prone to spoofing via remote access due to the lack of contact-based sensing. Gasti et al. developed an authentication technique for outsourced smartphones [23]. However, multimodal biometrics with face and voice used in this study are easier to compromise than other contact-based biometrics. In contrast, there are biometric signals potentially useful for wearable devices with spoofing-resistance such as wearable ECG [24,25], in-ear EEG [12], wearable MSP [16], and wearable vein sensor [26].

In this study, we propose a multimodal biometrics using our in-house wearable band integrated with MSP and ECG sensors. The proposed biometric system meets all the requirements for practical biometrics, including collectability by a single wearable device for multiple biometric sensors, performance by multimodal biometrics using MSP and ECG, and spoof-resistance by contact-based sensors for MSP and ECG. Since these sensors are cost-effective, multimodal biometrics with MSP and ECG have an advantage over other multimodal biometrics including iris or face that use relatively expensive image sensors for large-scale deployment [5]. In addition, the straightforward signal processing of MSP and ECG signals requires less computational resources than other biometrics relying on intensive image processing. In fact, all the algorithms developed in this study can be used in low cost wearable devices with limited power computation, memory, and battery capacity. In this regard, we opt to adopt simple authentication algorithms for ECG [14,15,24], and MSP [16], as well as their fusion.

Section 2 describes background information on ECG and MSP biometrics and multimodal biometric algorithms. Section 3 illustrates our integrated ECG and MSP wearable band with signal processing methods and user template guided filtering/normalization scheme for multimodal biometrics. Section 4 presents human experiments and authentication results. Section 5 and Section 6 finalize the article with discussion and conclusion.

## 2. Background

### 2.1. Multispectral Skin Photomatrix (MSP) Biometrics

Underneath the skin of the human forearm, especially nearby the wrist, lie many different types of anatomical parts such as bone, tendons, ligaments, median nerve, and blood vessels. Because these parts are positioned in a different layout and thickness, directly linked to the uniqueness of anatomical deployment in an individual, the anatomical structure inside the wrist can provide useful information for biometrics.

**Figure 1:**
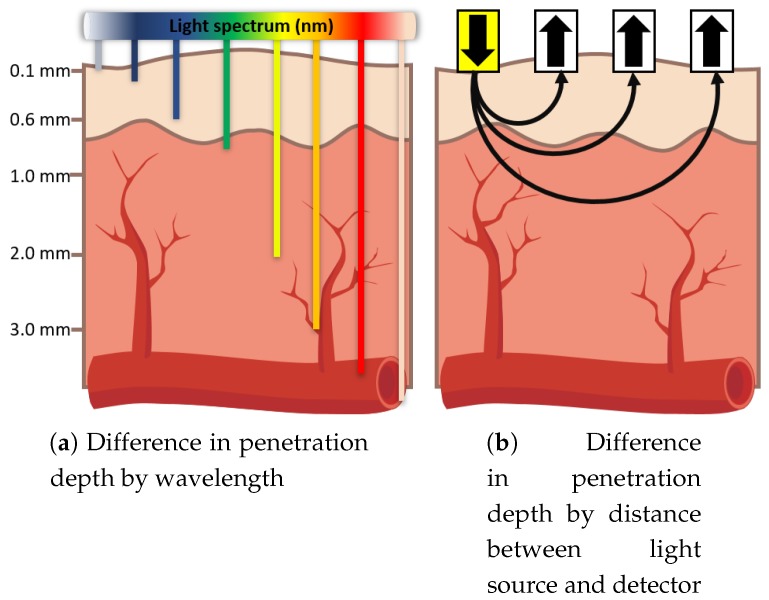
Illustrations of differences in penetration depth for Multispectral Skin Photomatrix (MSP) sensors by various wavelengths and distances between light source and detector.

In detail, these anatomical properties affect the pathway of light irradiated to the skin. When the photons penetrate into the human tissue, they take two actions depending on the internal structure of the tissue: absorption or diffuse reflection. Since the internal structure (such as location, size, density of blood vessels, tendons, and chemicals) is virtually unique to an individual, light intensities of absorbent and scattered reflection are also different across individuals. Moreover, the penetration depth of photon depends on its wavelength such as that we can measure the optical properties of various skin layers by modulating light wavelengths (Figure 1a).

The light emitted from a source in contact with the skin surface has a characteristic of spreading out in concentric circles, which implies that the penetration depth and intensity of light could vary with distance between the light source and the detector (Figure 1b). A detector far from the light source would obtain measurements reflecting those of the deeper layer. Therefore, we can obtain various optical properties by changing the distance between the light source and detector (Figure 1b).

Recently, a number of studies have demonstrated the biometric systems based on wrist vein patterns measured by optical sensors [9,26,27]. However, it may be also possible to explore distinct optical characteristics of a variety of wrist tissue structures in response to multiple light-wavelengths, which are likely to provide rich information for personal identification. The multispectral imaging technique that has been widely used for palm print, dorsal vein, and face recognition [28] can also be used for acquiring optical data for personal identification from the heterogeneous human wrist tissue structures. To test this hypothesis, we have developed a wrist-type of MSP biometric device that could acquire individual optical patterns of the wrist tissue [16]. Our previous study has successfully demonstrated a feasibility of using the MSP signal for personal authentication: 0.3% false acceptance rate (FAR) and 0.0% false rejection rate (FRR) with 21 subjects.

### 2.2. Electrocardiogram (ECG) Biometrics

**Figure 2:**
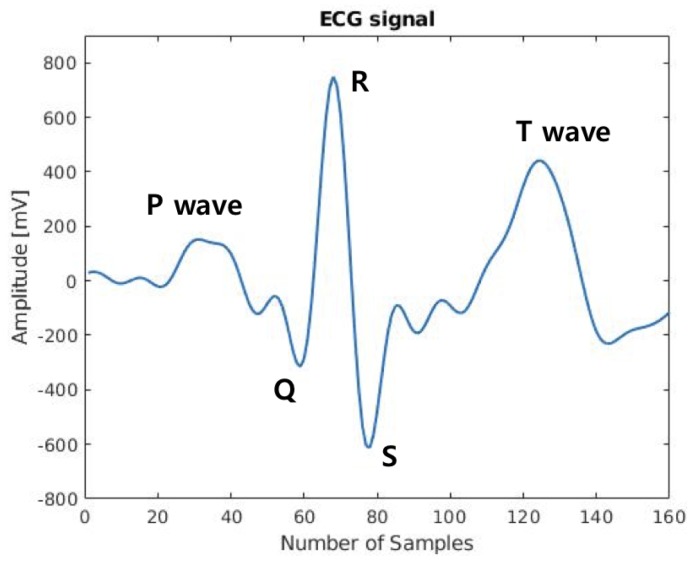
An Electrocardiogram (ECG) pulse measured by our in-house integrated wearable band with annotations for P-QRS complex-T.

ECG (Figure 2) is one of the biometrics that can naturally test the liveness of users [29]. ECG is also well suited for continuous verification over a long period of time [30,31]. Cancelable ECG biometrics have been investigated [32,33]. Using user template guided filtering (GF) has been investigated for improving the performance of challenging single pulse ECG authentication [14]. Intra-personal variability of ECG in biometrics has also been studied [13,34,35]. Constructing a user template from multiple sessions yielded promising performance [13]. Robust ECG features for heart rate (HR) variation can be selected and used for authentication [13,36,37]. Active QT interval correction methods for normalizing ECG signals yielded improved performances over no correction with [38] or without [15] knowing heart rate.

Medical-grade, heavy ECG signal acquisition system was one of the practical drawbacks for ECG biometrics [5]. However, recent advances in wearable ECG acquisition systems enabled the acquisition of ECG signals without requiring heavy systems [39,40,41,42]. Even though more sensors for ECG is beneficial to yield improved performance [43], most wearable ECG acquisition systems for a mobile environment adopt one-lead. ECG based authentication methods for mobile environment (e.g., limited computing power, limited memory) have been investigated in terms of hardware design [44] as well as lightweight software algorithms [24,45]. High authentication performances were achieved using wearable ECG sensors: 0.98% EER with chest wearable ECG with 30 subjects [45] and 0.9% EER with wrist wearable ECG with 15 subjects [24].

### 2.3. Distance Based Biometrics Authentication

It is common that the acquired biometrics signal is pre-processed ([46] for ECG and [16] for MSP) for user authentication to yield a feature vector *x*. Assuming *N* number of enrolled template vectors $x_{1}^{t},\ldots ,x_{N}^{t}$ and *M* number of current input vectors $x_{1}^{i},\ldots ,x_{M}^{i}$, conventional user authentication is done by measuring the distance between two sets:

(1) $$d\left(\left\{x_{1}^{t},\ldots ,x_{N}^{t}\right\},\left\{x_{1}^{i},\ldots ,x_{M}^{i}\right\}\right)\overset{\mathrm{reject}}{\underset{\mathrm{accept}}{≷}}\gamma ,$$

where *d* is a distance metric or classifier and γ is a threshold.

In ECG biometrics, it has been shown that a Euclidean distance based simple detector is actually a generalized likelihood ratio test (GLRT) detector if the pre-processed signal vector *x* is contaminated with independent and identically distributed (i.i.d.) Gaussian noise [15]. This method has been demonstrated to be effective for user authentication when proper performance improvement methods with mild computation increases are used together [14,15]. In this article, Euclidean distance with two averages of two sets was used for authentication as follows:

(2) $${∥t-s∥}_{2}\overset{\mathrm{reject}}{\underset{\mathrm{accept}}{≷}}\gamma_{E},$$

where $t=\sum_{j=1}^{N}x_{j}^{t}/N$, $s=\sum_{j=1}^{M}x_{j}^{i}/M$.

In MSP biometrics, the same Euclidean distance based approach was used for authentication. Light intensity features of different light sources (e.g., red and yellow) were concatenated into one feature vector that is similar to [16]. Then, the averages of feature vectors, *v* for enrollment and *z* for current input, were compared using Equation (2) with a threshold $\gamma_{M}$.

## 3. Methods

We developed a prototype of a wearable security band (WSB, model No: WSB-900) for authentication using biometrics. The WSB measured ECG and MSP signals simultaneously in a single portable device attached to the wrist of the user. It could either transmit the measured signals to other external systems or conduct an on-board authentication process directly within the device.

### 3.1. System Configuration of WSB

**Figure 3:**
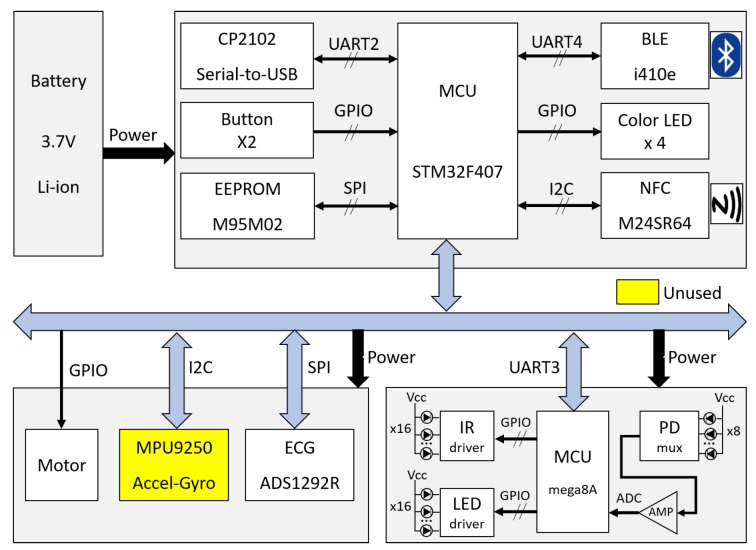
A block diagram of our in-house wearable security band (WSB).

Figure 3 illustrates the block diagram of the WSB. The WSB was equipped with a commercially available micro controller unit (MCU) chip (STM32F407, STMicroelectronics, Inc., Geneva, Switzerland) and three different telecommunication modules for USB 2.0, Bluetooth 4.0 and near-field communication (NFC) read/write. It also provided the user-interface (UI) with two input buttons, four color LEDs and a vibration motor controller. The mainboard of the WSB contained MCU, power supply, telecommunication modules, UIs and ECG processing module, whereas the MSP processing module was implemented inside the band-type band and connected to the mainboard via a wired link (Figure 3). The WSB also mounted an additional acceleration and gyro sensors that are not used in this study. A lithium polymer battery supplied the power to the WSB (3.7 V, 240 mAh), which was rechargeable by an integrated circuit (IC) (MCP73833) via USB connection to a PC.

### 3.2. ECG Signal Processing

**Figure 4:**
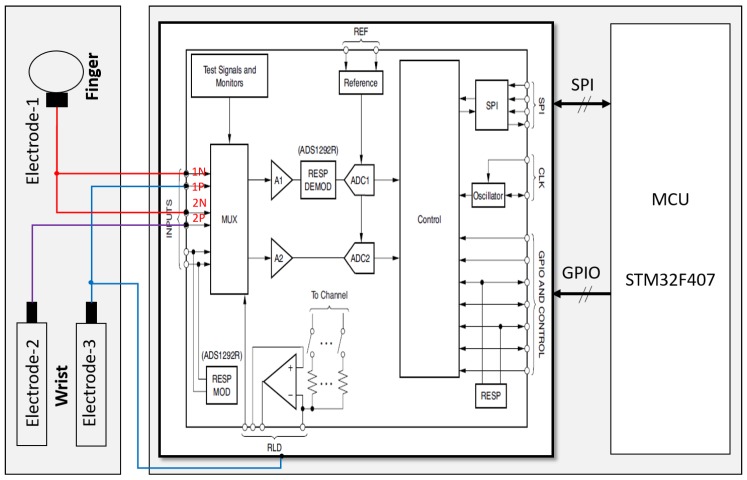
A block diagram of ECG signal processing module.

ECG signals were measured when the user wore the WSB on the wrist and touched the ECG electrode on the surface of the upper part of the WSB by the finger of the other hand. Three gold-coated aluminum ECG electrodes were attached to the WSB, one on the upper part (electrode-1) and the other two on the lower part (electrode-2 and electrode-3). When the user pressed electrode-1 on top of the upper part, electrode-2 and electrode-3 attached to the lower part automatically contacted with the skin of the wrist, starting to measure ECG signals. A commercially available chip (ADS1292R, Texas Instruments, Inc., Dallas, TX, USA) was used for the ECG signal processing. Electrode-1 was connected to the negative input-1 and the negative input-2 of the chip, electrode-2 was connected to the positive input-1, and electrode-3 was connected to the positive input-2, respectively. The sampling rate was 250 Hz. The outputs from positive input-1, and -2 yielded the raw ECG signals (Figure 4). These raw signals were transmitted to the Serial Peripheral Interface (SPI) Bus port of MCU and filtered by a lowpass filter with a cutoff frequency of 40 Hz followed by a 60-Hz notch filter. The filtered data were then stored in flash memory inside the WSB or transmitted to other systems via USB or Bluetooth links.

### 3.3. MSP Signal Processing

**Figure 5:**
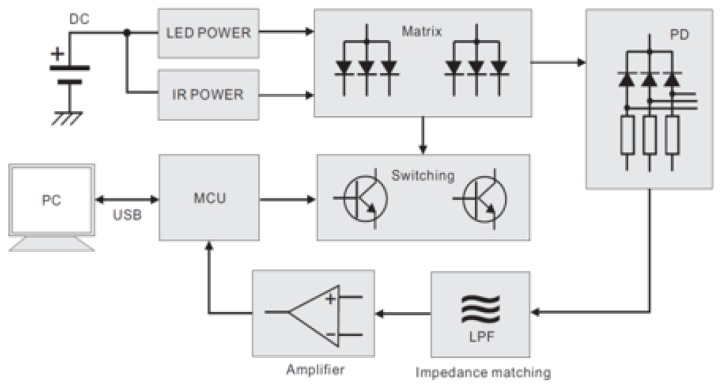
A block diagram of MSP acquisition module.

The MSP sensor module, attached to the lower part of the WSB to measure MSP data from the wrist, received the power from and sent digital MSP data to the main board via the wired connection. The serial communication protocol was used to transmit the MSP data between the module and the main board. The MCU in the main board either stored the MSP data to the local flash memory or transmitted them to an external device through USB or Bluetooth 4.0 links (Figure 5).

**Figure 6:**
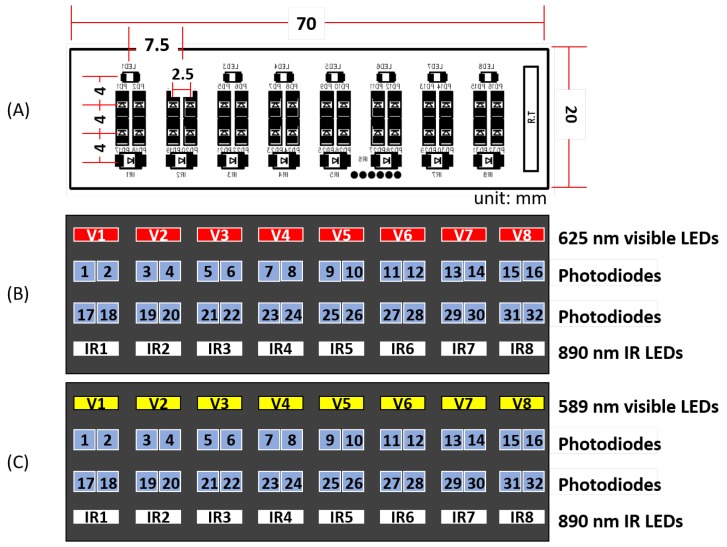
(**A**) The layout of LED arrays in an MSP module; (**B**) an MSP module equipped with eight red light LEDs and eight infrared light LEDs; and (**C**) an MSP module equipped with eight yellow LEDs and eight infrared LEDs. For both designs, 32 photodiodes (PD) are arranged between two layers of LEDs in a 2 × 16 matrix form.

The MSP module consisted of four subsystems, including the multi-wavelength light emitting diodes (LEDs, light sources), photodiodes (PD) arrays (light detection), an analog switching multiplexer, and amplifier circuits to drive the optical sensor. In this study, we used two types of MSP modules, each measuring the optical properties of either the red or yellow light wavelengths. The red-light MSP module consisted of eight 850-nm high power infrared LEDs (SFH 4655, OSRAM Opto Semiconductors GmbH, Regensburg, Germany) and eight 625-nm red LEDs (VLMR233, Vishay Intertechnology, Inc., Malvern, PA, USA) (Figure 6B). The yellow-light MSP module consisted of eight 850-nm high-power infrared LEDs (SFH 4655) and eight 589-nm yellow LEDs (VLMR233) (Figure 6C). The light detector part for both types was commonly composed of thirty-two silicon pin PDs (APISD 019-111-411).

The layout of the LED module together with PDs in the MSP arrays is illustrated in Figure 6. In the module, the LED arrays, light sources, were located in the outer area and the PD arrays, light detectors, were located in the inner area. This layout was designed to acquire the optical information simultaneously from both shallow and deep skin layers by using two different light pathways. Specifically, the PD detectors relatively close to the LED array measured short-distance photocurrents that were likely to contain optical responses of shallow layers in the wrist, whereas the detectors relatively far from the array measured long-distance photocurrents for optical responses of deep layers (see [16] for more detailed specification of the MSP sensor hardware).

**Figure 7:**
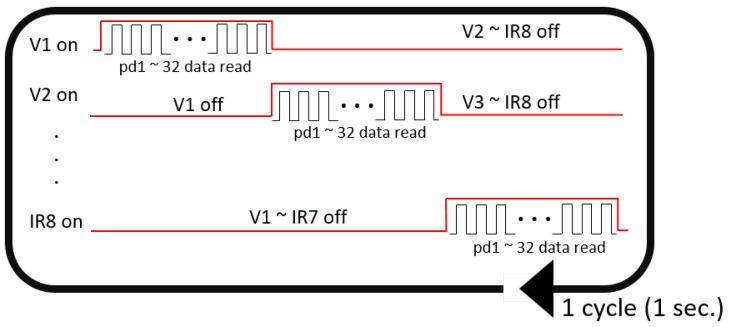
The operating process of the LED sources and Photodiode (PD) detectors for the single cycle of the MSP data acquisition. One of the 16 LEDs at the first visual channel (V1) begins to turn on and each of the 32 PDs detects optical signals in sequence. Then, after an interval, the next LED at the second visual LED channel (V2) turns on and the PDs detect the signals. This process is repeated for each of eight visual LEDs (V1-V8) and eight infrared LEDs (IR1-IR8). The entire process spans approximately 1 s.

The process of optical signal acquisition using the MSP module is illustrated in Figure 7. In a single cycle of signal acquisition, the PD detectors sequentially obtained optical signals from every LED source. Specifically, when one of 16 LED sources turns on, each of the 32 PD detectors turned on, received the optical signal and turned off in sequence, which as a whole took 1/16 s (LED ON). Then, the next LED source immediately turned on and the sequential signal acquisition by the PD detectors was repeated. Therefore, the entire period of detection for all the 16 LED sources spanned approximately 1 s. This cycle of signal acquisition was repeated four times in a single run of signal acquisition. Note that we obtained 32-channel light intensity data in response to two types of sources, eight visible (red or yellow, see Figure 6) and eight infrared (IR) light sources. Hence, we aggregated the light intensities detected within a cycle from all the sources of the same type (i.e., eight sources per type) into a single intensity value at each PD detector. This yielded a feature vector of 32 intensity values for each type of source per cycle. Hence, from a single run of MSP signal acquisition, we extracted four 32-dimensional feature vectors for each type of source.

**Figure 8:**
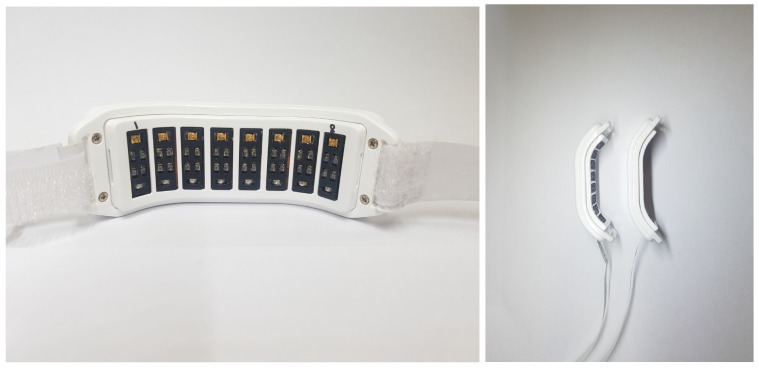
The optimized wrist surface type band by firmly attaching the PD array onto the wrist (**Left panel**). An examples of two bands with different curvatures optimized for: participants with small wrist (left in the **right panel**) and participants with normal or thick wrists (right in the **right panel**).

Besides the MSP module fully integrated in the WSB, we also developed a separate MSP sensor in order to improve contact with the wrist surface. As the MSP module was embedded inside the wrist-type band of the WSB, the contact of every single PD with the wrist surface could be affected by the way the user wore the WSB; it was possible that the PDs located on the edge of the band were slightly detached from the skin due to the curvature of the wrist in individuals. Hence, we created a separate MSP sensor to make MSP sensing more robust to variation of wrist curvature. For the sensor, we developed a wrist-type structure to firmly attach the PD array onto the wrist surface by optimizing the curvature of the structure (see Figure 8, right panel). The same MSP module as in the WSB was implemented inside the structure, but without the ECG module. A signal acquisition process with this MSP sensor was exactly same as that with the MSP module in the WSB (Figure 7).

### 3.4. User Template Guided Filter for MSP Signal

Guided image filter (GF) was originally proposed in computer vision and has yielded excellent performance in various applications such as denoising, artifact removal and upsampling [47]. Recently, Chun proposed to use a 1D GF in ECG authentication to yield improved performance [14]. This user template guided filter used the enrolled ECG template *t* as a guide signal to denoise the single pulse ECG input signal *s*. Since GF is essentially the local affine fitting of a guide signal to a noisy signal within local moving windows, this operation requires low computation complexity O(1) [47]. In this study, we extended this method to MSP. We denote this GF procedure for MSP signal as:

(3) $$\hat{v}=\mathrm{GF}\left(v;z\right),$$

where *z* is the enrollment MSP template (guided feature vector) and *v* is the incoming MSP feature vector to authenticate.

**Figure 9:**
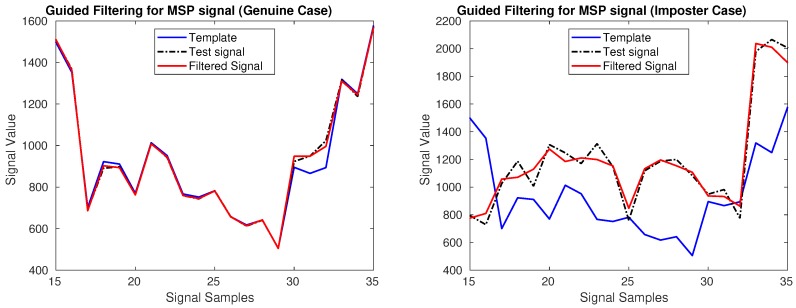
An effect of the application of a user template guided filter for MSP signal. The blue line shows the user template which is enrolled in the device. The black line represents the test signal, and the red line is guided filtering result with using template as a guide image.

Then, *z* and $\hat{v}$ are used for authentication instead of *z* and *v*. We adopted this method for performance improvement of distance-based MSP authentication. Figure 9 illustrates how the user template guided filter for MSP works for two cases. If $\hat{v}$ and *z* in Equation (3) are from the same person (genuine case, Figure 9 Left), the incoming MSP feature vector *v* is effectively filtered, near sample indices 17–20, and 30–33. If $\hat{v}$ and *z* in Equation (3) are from different people (imposter case, Figure 9 Right), then features in the incoming MSP signal *v* are destroyed substantially, for example, sample indices 17–20.

### 3.5. Single Threshold Multimodal Majority Voting

An information fusion strategy is one of the key factors to determine the performance of multimodal biometrics. There are largely three types of information fusion strategies in multimodal biometrics: decision level fusion, score level fusion and feature level fusion. Feature level fusion has yielded the best performance among three strategies in general [48]. However, it requires dealing with relatively high-dimensional feature vectors and fine-tuning classifiers with a fairly large biometric data set.

Decision level fusion is relatively easy to implement once individual classifiers exist and does not require other external data as far as individual classifiers do not need it. We conjecture that decision level fusion is more adequate for multimodal biometric wearable bands than other fusion strategies as it can alleviate a burden of loading external biometric data in the wearable bands.

One of the simplest decision level fusion algorithms is a majority voting algorithm [49,50]. When M number of modalities are given to determine acceptance or rejection, the final acceptance is decided only if the sum of each classifier’s acceptance decision is over half of M (Algorithm 1). Note that, since each modality involves different dimensionality and acquisition circumstances, it is common to use different thresholds for each modality classifier. However, we recognized that it was feasible to use a single threshold for multimodal MSP and ECG biometrics if each modality’s distance is normalized by its maximum distance. Hence, in this study, we set a single threshold for both MSP-, and ECG-based classifiers.

**Figure 10:**
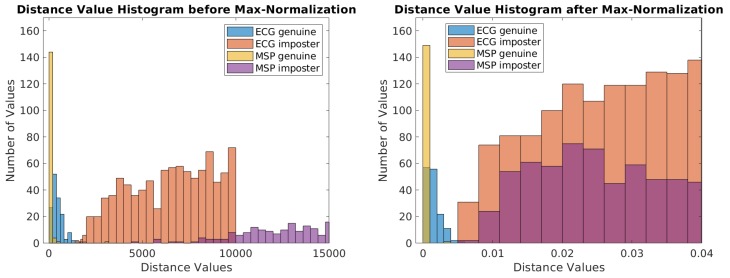
A comparison of the histograms of distance values before (**left**) and after (**right**) the normalization by the maximum distance. A distance value refers to an Euclidean distance between a user template and a tested biometric signal (ECG or MSP) of the genuine user or an imposter.

Figure 10 illustrates the effect of normalization by showing examples of two histograms of Euclidian distances before (left), and after normalization with the maximum values (right) in a representative subject. In the left panel of Figure 10, the distance values are distributed widely so that it is infeasible to use a single threshold for both modalities. On the contrary, in the right panel of Figure 10, since the distance values are normalized, it allows us to use a single threshold value for all modalities so that parameter tuning procedure can be simplified. This normalization procedure is similar to score normalization [51,52].
**Algorithm 1** Majority Voting Based Decision Level Fusion1:For the given threshold th, the number of classifier M and the feature vector c, the decision level majority voting accepts if
$$G\left(c\right)=\sum_{i=1}^{M}g_{m}\left(c\right)\overset{\mathrm{reject}}{\underset{\mathrm{accept}}{≷}}\left\lfloor M/2\right\rfloor ,$$
where ⌊·⌋ outputs the greatest integer that is less than or equal to c and $g_{m}$ is an individual classifier such that
$$g_{m}\left(c\right)=\left\{\begin{array}{c} 1,\;c>\mathrm{th},\\ 0,\;c<\mathrm{th}. \end{array}\right.$$

From Equation (2) with our distance normalization, it is possible to reformulate the majority voting algorithm for multimodal biometrics:

(4) $$G\left(c\right)=g_{1}\left(c\right)+g_{2}\left(c\right)\overset{\mathrm{reject}}{\underset{\mathrm{accept}}{≷}}\alpha ,$$

where c=[s;z],

$$g_{1}\left(c\right)=g_{1}\left(\left[s;z\right]\right)=d\left(t,s\right)\overset{\mathrm{reject}}{\underset{\mathrm{accept}}{≷}}\mathrm{th},$$

$$g_{2}\left(c\right)=g_{2}\left(\left[s;z\right]\right)=\tilde{d}\left(v,z\right)\overset{\mathrm{reject}}{\underset{\mathrm{accept}}{≷}}\mathrm{th}.$$

Note that α is 1 for a method that decides final acceptance when both modality classifiers accept (AND), and 0 for a method that decides final acceptance when at least one modality classifier accepts (OR). Note that the distances d(t,s) and $\tilde{d}\left(v,z\right)$ are normalized to have the same maximum distance.

## 4. Experimental Results

In this section, we elaborate on a personal authentication experiment to acquire ECG/MSP data using our in-house wearable wrist band, data processing, and experimental results.

### 4.1. Data Acquisition

**Figure 11:**
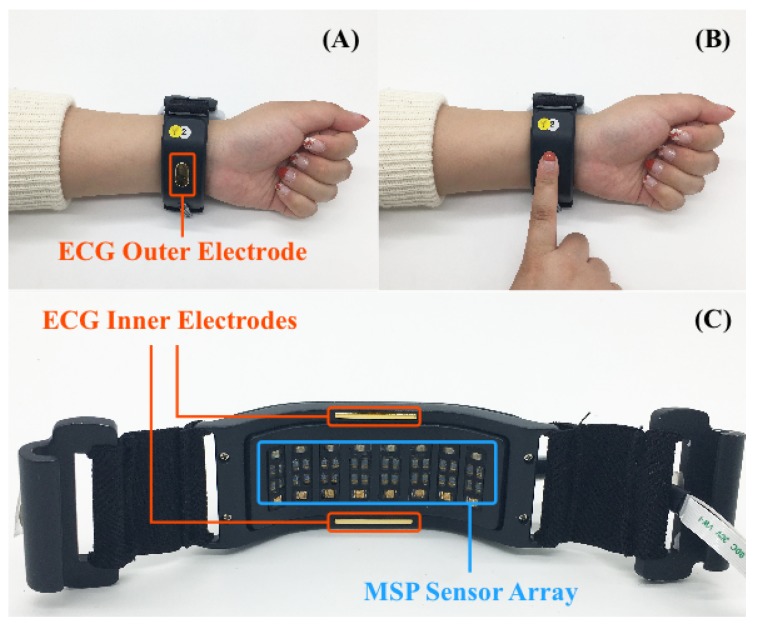
Snapshots of our in-house data acquisitions for ECG (**A**,**B**) and MSP (**C**). One wearable ECG sensor contacts the wrist of the left arm (**A**) and a subject must touch ECG sensors with their index finger of the right hand to acquire ECG data to measure the potential difference between left wrist and right finger (**B**). MSP sensor array is placed on the right wrist to measure the data (**C**).

Data acquisition was performed on single bespoke wearable devices, denoted hereafter as A, B, C and D. Devices A and B were the WSB incorporating both the ECG and MSP modules (see Section 3.3 and Figure 11C), whereas devices C and D were separate MSP sensors including only the MSP module (see Section 3.3 and Figure 8). Devices A and C implemented Red/IR MSP while devices B and D implemented Yellow/IR MSP. For ECG sensing, devices A and B featured three sensor electrodes, two on the inner side of the strap that made continuous contact with the skin of the wrist whilst the device was worn (see Figure 11C) and one on the outer side of the strap (see Figure 11A). During ECG acquisition, participants must touch the outward facing sensor with their index finger. This enabled the WSB to measure a potential difference between the wrist wearing the device and the index finger on the other hand, as shown in Figure 11B. The ECG sampling rate was 250 Hz and the ECG signal was filtered by a low pass filter with a cut off frequency of 40 Hz. MSP data acquisition was achieved by the sensor arrays in all devices, as shown in Figure 8 and Figure 11C. In all devices, the MSP sensor arrays were integrated into the wrist band such that it was in close proximity to the skin.

The ECG/MSP data for 150 participants were acquired from this study. All subjects in this study gave their informed written consent prior to participation in the study. The study was conducted in accordance with the Declaration of Helsinki, and the protocol was approved by the Institutional Review Board of the Ulsan National Institute of Science and Technology (UNISTIRB-16-01-G).

The study procedure for each participant was as follows. Firstly, the participant donned device A (Red/IR mode) on their left wrist. Secondly, the device was adjusted by the experimenter to ensure that the skin of the wrist made good contact with both the ECG electrodes and the MSP matrix. Thirdly, data capture took place in the following sequence:

(1) participants placed their right index finger on the outer facing ECG electrode,
(2) the ECG data stream was acquired for 30 s,
(3) participants removed their right index finger from the ECG electrode,
(4) participants then donned device C on their right wrist; MSP data from both devices A and C were simultaneously acquired from five runs of four cycles in the Red/IR mode (for approximately 20 s),
(5) participants removed devices A and C (Red/IR mode),
(6) participants donned devices B and D (Yellow/IR mode),
(7) MSP data from both these devices were acquired with five runs of four cycles in the Yellow/IR mode (for approximately 20 s),
(8) Finally, participants removed the devices and data capture was finished.

### 4.2. Data Pre-Processing and Evaluation Criteria

A minute-long ECG data was divided into multiple ECG single through the following procedure: (1) baseline correction using a band-pass filtering, (2) R-peak detection using the Pan-Tompkins algorithm [53], and (3) extraction of individual P-QRS-T fragments with the length of 160 samples (0.64 s), which were in between −67 samples before and +92 samples after the R-peak. We extracted 36 pulses from the ECG data and repetitively averaged two successive pulses, generating 18 averaged pulses. These averaged pulses were grouped into six records each containing three successive averaged pulses. Thus, the structure of the final ECG data was 150 (number of subjects) × 6 (number of records per subject) × 3 (number of pulses per each record) × 160 (number of ECG samples per pulse).

A single cycle of MSP data acquisition generated a 32-D red feature vector plus a 32-D IR feature vector from the Red/IR mode or a 32-D yellow feature vector plus a 32-D IR vector from the Yellow/IR mode, respectively, and a total of 20 cycles were operated for each mode. Then, we concatenated all the feature vectors per cycle from each mode into a single 128-D feature vector (consisting of 32 Red, 32 Yellow and 64 IR light intensities). From these 20 feature vectors, we randomly selected 18 and grouped them into six records each containing three feature vectors, in order to be consistent with the ECG case. Thus, the structure of the final MSP data was formed as 150 (number of subjects)× 6 (number of records per subject)× 3 (number of vectors per record)× 128 (MSP features per vector). Taken together, six records of ECG and MSP were constructed with three ECG pulses and three MSP feature vectors per record for each subject. In all the authentication procedures of this study, an enrolled template was constructed by averaging six feature vectors (i.e., 6 ECG pulses) from two records (i.e., N=6, in Equation (2)), and an authentication input was constructed by averaging three vectors (i.e., three pulses) from one record (i.e., M=3, in Equation (2)).

For each subject, we chose two out of six records for the enrollment to form a personal template and conducted the authentication test on the remaining four records, together with 596 records of others (four records of 149 subjects). This test was repeated 15 times: for all possible combinations of two out of six records. Then, we repeated the same authentication procedure for all the subjects.

Our proposed authentication method was based on the Euclidean distance between the enrolled biometric template and the testing biometric data for each biometric modality as described in Equation (2). All the Euclidean distances for ECG and MSP were normalized such that the maximum distance in ECG and the maximum distance in MSP were equal to each other. This procedure enabled us to use the same threshold for both ECG and MSP biometrics and to simplify their fusion.

Several performance evaluation criteria were used for the proposed ECG/MSP multimodal biometrics. FAR (False Acceptance Rate) is the ratio of the number of times when an actual imposter was predicted as genuine over the number of all the actual imposter cases ($\mathrm{FAR}=\frac{\mathrm{False}\mathrm{Accept}}{\mathrm{False}\mathrm{Accept}+\mathrm{True}\mathrm{Reject}}$). FRR (False Rejection Rate) is the ratio of the number of times when an actual genuine was predicted as an imposter over the number of all the actual genuine cases ($\mathrm{FRR}=\frac{\mathrm{False}\mathrm{Reject}}{\mathrm{False}\mathrm{Reject}+\mathrm{True}\mathrm{Accept}}$). EER was obtained by finding a threshold where FRR=FAR:=EER. PD.1 was the detection probability at FAR=1%. As a fixed threshold, th determines FAR for the same threshold level, the higher detection probability a biometric method yields, the better performance it achieves. We also used the ${\mathrm{FRR}}^{0}$ for comparative performance evaluation under FAR=0%, indicating that the lower reject ratio represents the better performance.

### 4.3. Proposed Methods Description and Authentication Results

Table 1 shows the performance evaluation result of the proposed multimodal biometric authentication using the ECG + MSP integrated in WSB. It also shows the performance result using ECG in the WSB and the separate MSP sensor with better contact. Note that applying the majority voting (MV) method for two modalities can be conducted in two different ways: in one way, final acceptance is decided when both modality classifiers accept (AND), and, in the other way, final acceptance is decided when at least one modality classifier accepts (OR). Note that the same distinction algorithm was used for both ECG and MSP signals. In addition, this algorithm was applied to both cases when two signals were measured within a single integrated wristband (ECG + MSP integrated in WSB) or when they were measured separately (ECG + separate MSP sensor).

For both cases of different MSP setups, our multimodal biometrics with the MV based on OR fusion yielded superior performance to the MV based on AND fusion: EER=0.2%,PD.1=100% and EER=0.1%,PD.1=100% using the WSB integrated with ECG and MSP and the combination of ECG and a separate MSP sensor, respectively. EER of 0.1% is comparable to the state-of-the-art performance of recent multimodal biometrics [22]. In addition, the combination of ECG with a separate MSP sensor (ECG w/separate MSP sensor in Table 1) for better contact remarkably improved ${\mathrm{FRR}}^{0}$ to be as low as 0.2%. Moreover, our proposed method has advantages such as good circumvention property (stealing ECG or MSP would be more difficult than face or voice information in [22]).

**Figure 12:**
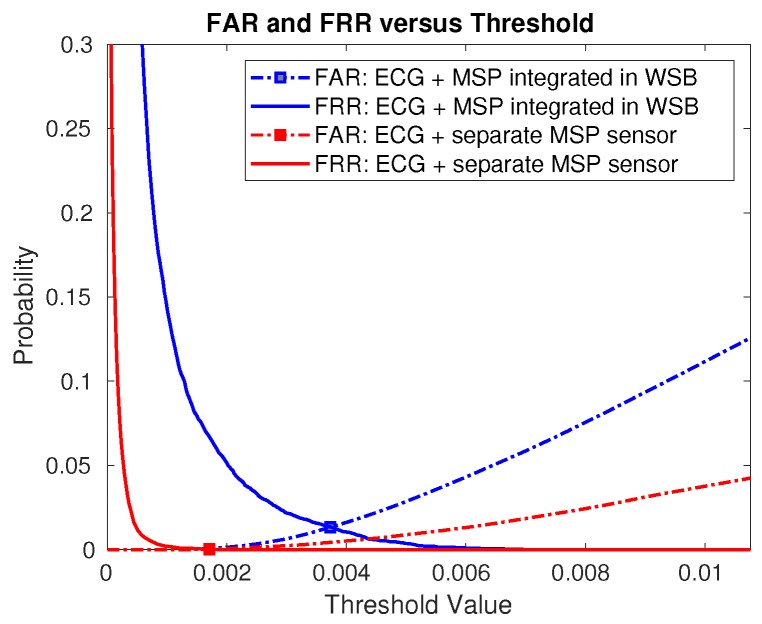
False Acceptance Rate (FAR) and False Rejection Rate (FRR) graphs for different threshold values for proposed multimodal biometrics methods: ECG + MSP integrated in Wearable Security Band (WSB); and ECG and MSP separately measured for better contact (ECG + separate MSP sensor), respectively.

We also examined the effect of GF on performance by analyzing authentication outcomes: without applying guided filter (no GF), partially applying guided filter on ECG signal only (GF${}_{\mathrm{ECG}}$), and applying guided filter on both modalities (ECG and MSP, GF${}_{\mathrm{ECG},\mathrm{MSP}}$). By applying GF to each modality, the performance in most cases have been enhanced. Specifically, under an extremely difficult situation (i.e., ${\mathrm{FRR}}^{0}$), GF showed a remarkable boost in performance (see Table 1, MV(OR) result). Figure 12 illustrates changes in FAR and FRR with various thresholds. Note that FAR and FRR are non-decreasing and non-increasing, respectively, for an increasing threshold.

## 5. Discussion

The proposed wearable wrist band integrated with MSP and ECG sensors enabled multimodal biometrics in a single portable device and demonstrated a feasibility to provide strong security with advantages in collectability (single wearable wrist band with multimodal biometric sensors), performance (multimodal fusion algorithm with MSP and ECG), and circumvention (MSP and ECG data that are not easy to steal remotely).

In the present study, our proposed wearable wrist band with two sets of sensors collects biometric signals and then the authentication process is conducted separately in a computer. Even though this scheme is still useful as it is, it will be more widely applicable and more secure if the authentication process can be performed inside the band. It is potentially possible in our current algorithm due to its simplicity. Our proposed multimodal biometrics method exploits a simple Euclidean distance measure for individual biometrics as well as a simple fusion scheme based on majority voting. These algorithms only require lightweight computation so that they are suitable for wearable devices with limited computation power. Even though there are many computationally powerful wearable devices these days, heavy computation still requires high energy consumption. Thus, our proposed algorithms have an advantage over other algorithms in terms of computational loads.

For the development of multimodal biometrics in the wearable band, we conjecture that the wearable band should allow limited access to others’ biometric data. Most state-of-the-art biometrics methods yield excellent results by exploiting high-dimensional feature spaces and sophisticated decision rules based on large amounts of training data (e.g., see [13]). However, our proposed method only requires the computation of a few parameters (distances d(t,s), $\tilde{d}\left(v,z\right)$ and a threshold th). Our preliminary study (not shown here) suggested that weights in multimodal fusion could be obtained from a small number of sample data so that it may be possible to fix these values. In that case, no additional biometric information will be required for a stand-alone wearable band equipped with sensors and authentication processing. Further investigation will be necessary to confirm this conjecture.

Odinaka et al. showed that ECG biometrics could yield up to 0.03% EER, but with 64 ECG pulses for the user template generation and another 64 ECG pulses for authentication which were acquired on the same day per subject using medical grade ECG acquisition systems [13]. They also showed that the same authentication method could yield up to 0.38% EER with 32 pulses. In ECG, using more pulses implies longer acquisition time for authentication-e.g., acquiring 64 pulses would take approximately 1 min. In this study, only averaged three ECG pulses were used for fast response authentication and by fusing it with MSP efficiently, the proposed method yielded up to 0.1% EER, which is much better than ECG biometrics using 32 pulses and other subjects’ data for sophisticated classifer training. Our proposed methods have advantages of both high performance and practical response time over other state-of-the-art methods.

Even though many promising results were presented in this article, there are also limitations. All the biometric data were acquired on the same day, so a possible biometric signal variation over time in individuals was not tested. However, there have been many works to deal with ECG signal variation over time for authentication. For instance, Chun proposed methods to deal with short-term and long-term ECG signal variations for high performance ECG based authentication [15]. Incorporating signal variation models in the proposed methods and validating them with extensive biometric data sets can be an important next step of the work.

Furthermore, we will pursue generalizing proposed wearable wrist band-type multimodal authentication system for practical usage. Also, in this study, we were only concerned with normal sinus ECG rhythms to test our integrated system for a general population. However, it would be also important to consider applying our system to specific cases such as the users with arrhythmia or vertical heart. In addition, there is a possibility for changes in ECG signals due to various status of subjects such as heart rate or orientation of hearts. In the present study, we controlled the orientation of the heart by letting the subjects stand still during measurements. In addition, we also controlled the heart rate of the subjects as there are many studies showing that ECG signal changes, especially T-wave changes, due to changes in heart rates (e.g., running or standing steady) [15,35]. Since our proposed algorithm leverages the shape of ECG signal itself, it may need to be tested our algorithm for the identification of those cases as well in the follow-up studies.

In this article, we performed authentication in two ways of measuring MSP signals, one fully integrated in the WSB and the other built separately to enhance contact with the skin. Since the MSP signals can be influenced by ambient lights, it is emphasized in this study that the photomatrix system was firmly attached to the skin to prevent influences of ambient lights. To achieve this, we fabricated MSP modules with various curvatures and tested a fit of each curvature to the wrist surface. It allowed us to find the best fit between the curvature and the wrist surface so that the MSP signals were hardly affected by external lights. In addition, we asked the user not to move during the measurements to minimize motion artifacts. In doing so, we have not observed significant influences of potential artifacts. Otherwise, it is also possible for MSP signals to be influenced by other motion artifacts generated by placing a finger on the device when the ECG is measured. To avoid this, we can start to measure the data after the finger is positioned and make sure that users do not move after touch, so that ECG and MSP will not be affected by motion artifacts. However, in the present study, we have adopted the sequential measurement method instead of the simultaneous measurement in consideration of a possibility that the pressure generated from the finger may affect the MSP signals.

On the other hand, there might be an effect if the user puts the MSP sensor in another position. When photons emitted from the LED meet components in the skin tissue, they are absorbed or diffuse-reflected. A unique MSP pattern is generated from the behaviors of these photons. If the user places the device at another position, the internal structure of the skin (bone, ligament, collagen, etc.) that the photons meet in the skin will change. As such, the angle and depth of diffuse-reflectance become different, which can result in changes in the MSP pattern. This would lead to an increase in FRR by more failure to identify the user.

The results showed substantial improvement of performance using the separate MSP sensor, demonstrating the importance of reliable contact between the MSP sensors and the wrist skin. From this, our follow-up research is developing an advanced version of the WSB with the new structure that ensures firm contact of the MSP sensor. Lastly, we additionally examined the effect of the number of users on multimodal authentication performance. We found that the performance of authentication using ECG + MSP integrated in WSB started to decrease in terms of ${\mathrm{FRR}}^{0}$ score when 50 or more users were tested. In contrast, the performance using the ECG + separate MSP sensor did not degrade much as the number of users increased. To generalize this effect, we will test our methods with a much larger number of subjects.

## 6. Conclusions

We developed an integrated wrist band with MSP and ECG sensors for multimodal biometric authentication. Our proposed device has advantages such as good collectability due to single band, excellent performance due to multimodal biometrics and powerful fusion algorithm, and good circumvention properties due to contact based MSP and ECG sensors that are not prone to be compromised remotely. Our experiments with 150 healthy subjects using our in-house integrated MSP-ECG wearable sensors yielded up to 0.1% EER, which is comparable to previous state-of-the-art multimodal biometrics, while our proposed method has potential advantages for practical usage in collectability and circumvention.

## Figures and Tables

**Table 1:** Performance summary for multimodal biometric authentication results using Electrocardiogram (ECG) and Multispectral Skin Photomatrix (MSP).

		Modality					
		ECG + MSP Integrated in WSB			ECG + Separate MSP Sensor		
		EER [%]	PD.1 [%]	${\mathrm{FRR}}^{0}$ [%]	EER [%]	PD.1 [%]	${\mathrm{FRR}}^{0}$ [%]
MV(AND)	no GF	2.8	86.3	17.9	0.3	100	2.8
	GF${}_{\mathrm{ECG}}$	2.8	85.0	14.0	0.2	100	1.9
	GF${}_{\mathrm{ECG},\mathrm{MSP}}$	2.6	85.7	16.4	0.2	100	2.4
MV(OR)	no GF	0.4	99.9	41.0	0.1	100	0.3
	GF${}_{\mathrm{ECG}}$	0.2	100	45.9	0.1	100	0.3
	GF${}_{\mathrm{ECG},\mathrm{MSP}}$	0.2	100	21.0	0.1	100	0.2

Majority Voting (MV), Guided Filter (GF), Equal Error Rate (EER), Detection Probability at FAR=1% (PD.1), False Rejection Rate at FAR=0% (${\mathrm{FRR}}^{0}$).

## Abbreviations

The following abbreviations are used in this manuscript:

ECG	Electrocardiogram
EEG	Electroencephalogram
EER	Equal Error Rate
FAR	False Acceptance Rate
FRR	False Rejection Rate
${\mathrm{FRR}}^{0}$	False Rejection Rate at FAR=0
GF	Guided Filter
GLRT	Generalized Likelihood Ratio Test
HR	Heart Rate
MSP	Multispectral Skin Photomatrix
MV	Majority Voting
PD	Photodiode
PD.1	Probability of Detection at FAR=1%
WSB	Wearable Security Band
